# Expanding accessible chemical space for fragment-based enumeration by orders of magnitude through optimization of the Chemically Reasonable Mutations (CReM) framework

**DOI:** 10.1186/s13321-026-01265-8

**Published:** 2026-08-01

**Authors:** Shaojin Hu, Qinyu Chen, Yinhui Yi, Paul Pillot, James Xu, Evgeny Gutkin, Abir Ganguly, Albert C. Pan

**Affiliations:** TandemAI, New York, NY 10036 USA

## Abstract

**Abstract:**

The size of synthetically accessible small molecule chemical space has rapidly grown to billions, and even trillions, of molecules, unlocking tremendous new opportunities for drug discovery. Fully leveraging these ultralarge chemical libraries, however, will strain existing computational tools, requiring novel approaches to efficiently search and process this vast space. The Chemically Reasonable Mutations (CReM) method is a powerful approach for the generation of novel and synthesizable molecules using precomputed fragment libraries. While expanding the size of these libraries could greatly enhance CReM’s ability to afford higher quality ideas, improving the scalability of the current framework is needed to take advantage of these larger libraries in practice. Here, we introduce an optimized fragment library framework, oCReM, that eliminates redundancies in the fragment database along with modifying corresponding core functions in CReM. oCReM affords a reduction of storage requirements and query times by significantly over 50%, enabling the use of ultralarge fragment libraries on the scale of tens of billions of fragments and dramatically expanding the size of chemical space available for generative design. We show that the molecules generated using the larger fragment libraries enabled by oCReM are more diverse, drug-like, and potent compared to those generated using a smaller library.

**Scientific Contributions:**

We introduce oCReM, an open-source software framework that broadens the scope of the Chemically ReasonableMutations (CReM) method by enabling the use of billion-level fragment databases derived from ultralarge chemicallibraries. oCReM achieves this scalability through an optimized fragment database architecture and associated queryprotocols, signifi cantly reducing both storage requirements and query times. We demonstrate that expanding thefragment base leads to quantitative improvements in the diversity and quality of generated molecules, highlightingoCReM’s potential to advance large-scale, fragment-based molecular design.

**Supplementary Information:**

The online version contains supplementary material available at https://doi.org/10.1186/s13321-026-01265-8.

## Introduction

The size of drug-like chemical space has been estimated to be on the order of $10^{33}$ molecules [1], a size which is virtually impossible to explore with traditional workflows. Generative approaches [2] have emerged as promising ways forward for searching this vast space in a high throughput and systematic manner. A major challenge for generative approaches, however, is ideating truly novel molecules while ensuring their chemical validity and synthetic feasibility.

The Chemically Reasonable Mutations (CReM) framework [3] is a powerful and flexible generative approach that creates novel molecules from input structures using a database of interchangeable fragments. Given a small molecule, CReM generates new ideas by swapping substructures of the molecule with fragments which exist in a similar chemical context. The similarity of the context can be adjusted by setting a context radius: the larger the radius, the larger the context match that is required for a swap. In this way, chemically valid molecules are generated by design, and synthetic feasibility can be tuned by the size of the context radius. More recently, the CReM approach has been extended to enumerating molecular structures that conform to three dimensional pharmacophore models (CReM-pharm) [4].

The novelty and diversity of compounds generated by CReM is directly related to the novelty and diversity of the molecules in the fragment database. The original CReM release had a database based on ChEMBL22 [5], a manually curated collection of bioactive molecules with drug-like properties of approximately 1.56M molecules. The CReM repository (https://www.qsar4u.com/pages/crem.php) currently offers several fragment libraries, albeit of relatively modest size, derived from the ChEMBL22, ChEMBL23, and Enamine databases through the application of specific structural and property-based filters. In recent years, collections of synthetically accessible small molecules have continued to grow rapidly, with some like PubChem [6] and Enamine REAL [7] reaching 120M and 10B molecules in size, respectively.

Creating fragment databases from these much larger libraries would significantly boost the generative power of CReM. Internally, for example, we have greatly expanded our fragment database by building an ultralarge CReM-compatible fragment library, TandemDB, assembled from five widely used chemical databases: ChEMBL (1.7 million molecules) [8], Enamine (48 million) [9], SureChEMBL (22.6 million) [10], PubChem (108 million) [11], and ZINC20 (897 million) [12]. In the case of Enamine, we considered a structurally diverse, drug-like subset of the entire library, and for ZINC20, we considered the “Wait Ok” dataset that contains all molecules that are likely to be purchasable within some reasonable timeframe. While TandemDB dramatically increased our coverage of chemical space, it also introduced a significant challenge to our computational infrastructure. The combined total of 988 million source molecules produced a database with more than 40 billion fragments and a storage footprint that exceeded 10 TB, making efficient querying difficult and impractical.

Here, we introduce a new framework for CReM fragment databases, oCReM (optimized CReM), that significantly reduces disk storage requirements and increases fragment query speed, making it feasible to use ultralarge libraries for generating novel molecules with unparalleled structural diversity. The remainder of the paper is structured as follows: we begin by presenting the original CReM framework, and then detail our optimized oCReM framework, highlighting its key distinctions from the original implementation. We then provide a direct comparison of library sizes and enumeration capabilities between the optimized and original versions. Finally, we present case studies that demonstrate the benefits of accessing the vastly expanded chemical space enabled by this optimization.

## The original CReM framework

In the original CReM framework, the fragment library is stored as a single table in an SQLite database with the following components (Table 1):

- core_smi: the SMILES representation of the fragment with its attachment point(s).
- env: the chemical environment of the fragment, defined by the topological distance (i.e., the number of bonds) from its attachment point(s). Fragment environments are recorded for context radii ranging from 1 to 3.
- core_sma: the SMARTS pattern that integrates core_smi with its environment.
- core_num_atoms: the number of atoms in the fragment.
- dist2: the topological distance between two attachment points in the fragment, and
- freq: the frequency of occurrence of a given combination of env and core_smi in the library

**Table 1:** Example of a CReM fragment library

env	core_smi	core_num_atoms	core_sma	dist2	freq
*#C-[*:1]	BrC#CC1([*:1])C2(CC2)C12CC2	10	[Br]-[C]#[C]-[C]1(-[C:3]-#[*:2])-[C]2(-[CH2]-[CH2]–2)-[C]−12-[CH2]-[CH2]−2	0	1

A given query molecule is first fragmented at the specified attachment point(s) to generate the fragment SMILES (query_smi) and the corresponding chemical environments (query_env) at those sites. The chemical environment captures the local molecular topology within a defined topological radius around each attachment point. Fragment SMARTS patterns are then constructed by combining each query_smi with its query_env, preserving fully the specified connectivity throughout the fragment except at the attachment site, while explicitly encoding the chemical environment of the attachment point(s). The CReM library is then searched for fragments with environments (env) that match query_env. For the matching fragments, a SMARTS-based transformation is carried out to replace query_sma with core_sma, producing new molecules (Algorithm  1).

**Algorithm 1:**
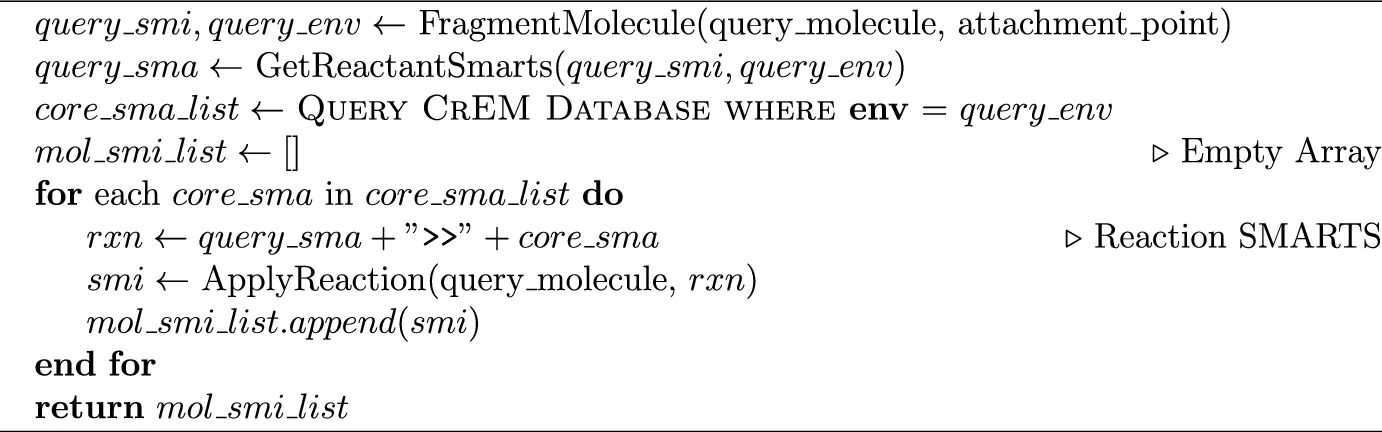
SMARTS-based reaction in CReM to replace a query molecule fragment with a chemically compatible fragment

This framework supports both R-group enumeration using the *grow_mol* function when a single query_env is defined from the query_smi, and scaffold_hopping using the *mutate_mol* function, when multiple query_env are defined. In this way, the approach enables a systematic exploration of chemical space with flexible control over molecular diversity, novelty, and synthetic feasibility.

## oCReM: an optimized CReM framework

Major contributors to the fragment library size are the core_smi and core_sma fragment entries, each of which can be hundreds of characters. An alternative approach would be, for a given query molecule, to identify its core (query_smi), R-group(s), their chemical environment(s) (query_env), and attachment point(s). The query_env can then be used to find matching core_smi, which can be directly attached as R-groups to generate new molecules (Algorithm 2).

If the query molecule has multiple R-groups, its specific chemical environments can guide correct attachment placement. This strategy eliminates the need to store fragment SMARTS (core_sma), reducing by as much as 50%, the data that needs to be stored in the tables, excluding the table indices.

**Algorithm 2:**
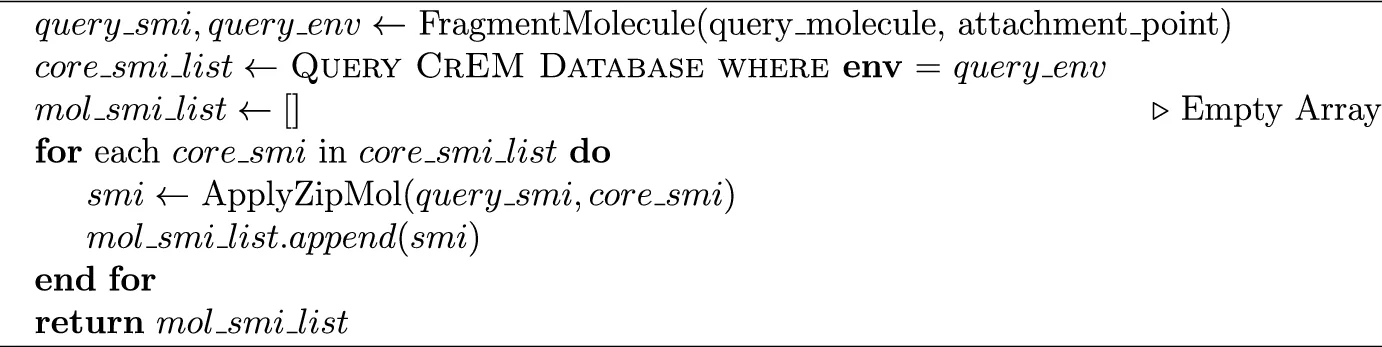
Strategy for directly attaching fragments to a query molecule core, without using a query or replacement fragment SMARTS

In CReM, a single fragment may correspond to multiple chemical environments, and each environment can, in turn, match several fragments. In the original fragment library format, all such combinations are stored as a flattened list, leading to redundancy in both the env and core_smi columns. This redundancy becomes more pronounced when the libraries are built from multiple chemical databases. A more efficient approach would be to maintain separate, de-duplicated, and indexed tables of unique env and core_smi, along with a mapping table that defines their relationships (Fig. 1). This new database schema would further reduce the overall size of the fragment library. Together, these optimizations significantly reduce the size of the fragment library while also improving the efficiency of library queries.

**Fig. 1:**
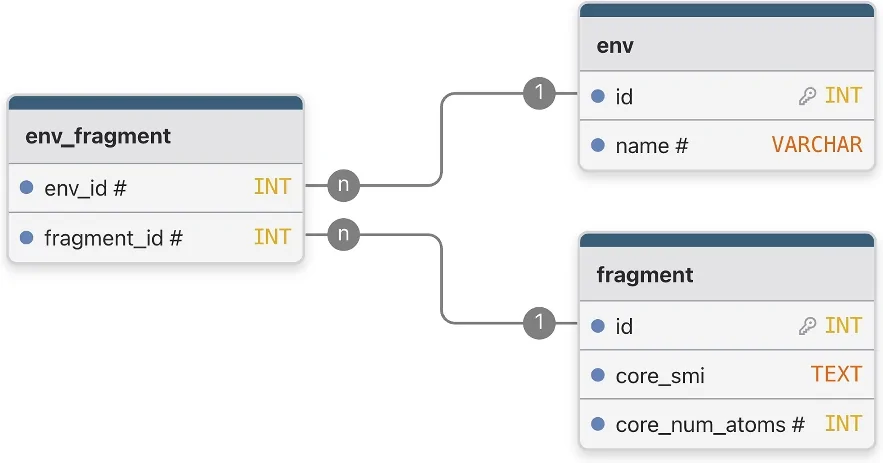
Schema of the oCReM fragment libraries. Indexed fields are denoted with a # sign. Symbols **1** and **n** indicate a one-to-many relationship between a primary key and a foreign key in the env_fragment table

## Performance comparison between the CReM and oCReM frameworks

To demonstrate the advantages of this optimized implementation of CReM, we consider fragment libraries derived from ChEMBL22 that were available as part of the original CReM distribution (https://www.qsar4u.com/pages/crem.php). CheMBL22 contains 1.7 million molecules and the CReM-compatible fragment library for ChEMBL22, in its original (SQLite DB) form, contains 110 million items and has a size of 30 GB. Our optimized version of this library has a size of 15.1 GB, a reduction of 51% (Table 2).

We note that we chose PostgreSQL over SQLite internally to support our ultralarge fragment libraries due to PostgreSQL’s superior scalability, advanced indexing capabilities, and robust concurrent access support. Unlike SQLite, which is limited by its single-writer model and file-based architecture, PostgreSQL offers a client–server model that can efficiently handle complex queries and large volumes of data. Its extensible design, support for parallelism, and rich feature set make it better suited for managing the scale and performance demands of our optimized fragment enumeration framework. The ChemBL22 fragment library, in its optimized form in PostgreSQL, has a similarly reduced, 14.6 GB footprint (Table 2).

**Table 2:** ChEMBL22 fragment library sizes in the oCReM framework in SQLite and PostGRes formats

Table	SQLite DB		PostgreSQL DB	
	Size (GB)	Num entries	Size (GB)	Num entries
env	0.7	4,808,507	1.3	4,808,507
fragment	7.1	24,111,165	7.3	24,111,166
env_fragment	7.3	99,210,400	6	99,351,700
Total	15.1	128,130,072	14.6	128,271,374

To demonstrate the efficiency gains enabled by oCReM in a practical drug discovery setting, we carried out two molecular enumeration case studies: an R-group enumeration on the purine-based CDK2 inhibitor NU6094 [13] using the *grow*_*mol* function, and a core hop enumeration on MRTX0902, an inhibitor of the protein-protein interaction (PPI) of SOS1:KRAS [14], using the *mutate*_*mol* function (Fig. 2). The enumeration statistics are summarized in Table 3. In both cases, oCReM reproduced the same set of molecules as CReM, confirming functional equivalence. The optimized version, however, outperformed the original in speed, achieving a 30–67% improvement in the R-group enumeration speed and similarly large speed-ups in the core hop enumeration.

**Fig. 2:**
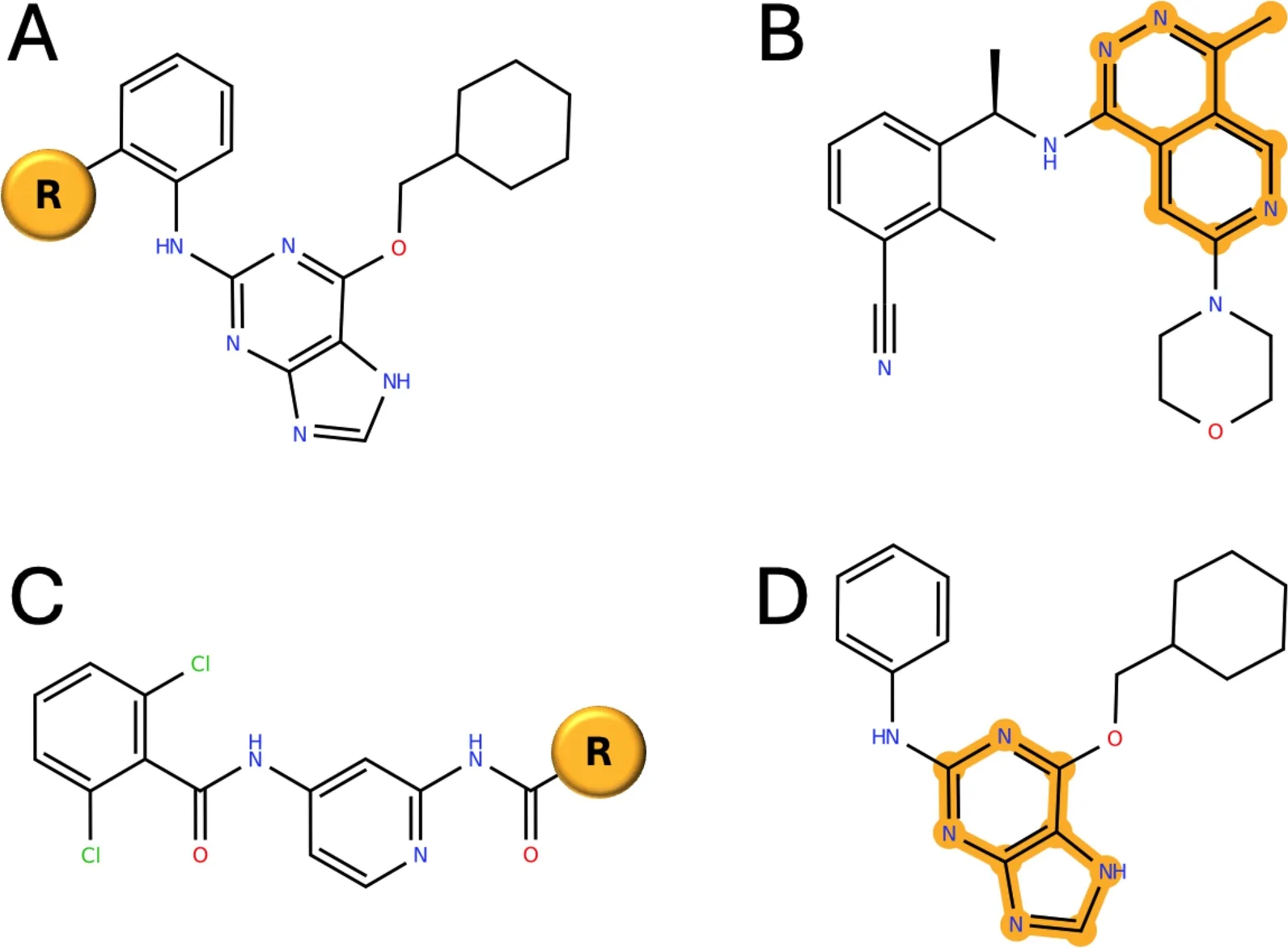
Molecules selected for enumeration using CReM and oCReM. Panels **A** and **D** show the CDK2 inhibitor NU6094 scaffold, **B** shows the SOS1:KRAS PPI inhibitor MRTX0902, and **C** shows a TYK2 inhibitor scaffold. R-group enumeration is performed for **A** and **C**, whereas core hop enumeration is applied to **B** and **D**. In each structure, the enumeration site or core is highlighted in gold

**Table 3:** Comparison of R-group and core-hop enumeration metrics between CReM and oCReM.

Mol	R	N mol		Time (s)			Speed gain (%)	
		CReM	oCReM	CReM	SQL	PG	SQL	PG
CDK2	1	458,401	458,401	1088	706	765	35.1	29.6
	2	91,736	91,736	194	124	132	36.1	31.9
	3	2470	2470	9	3	3	66.7	66.7
SOS1	1	19,221	19,221	34	24	22	29.4	35.3
	2	1764	1764	7	2	2	71.4	71.4
	3	151	151	1	<1	<1	–	–

The column labels SQL and PG refer to oCReM fragment databases in SQL and PostGresSQL formats. All enumerations were carried out on a single Intel(R) Xeon(R) CPU E5-2678 v3 @ 2.50GHz CPU. Speed gain (%) is computed as $\frac{Time^{CReM} - Time^{oCReM}}{Time^{CReM}}$

The advantages of oCReM become more pronounced with increasing size of the fragment library. As mentioned earlier, we assembled a large fragment library in house, TandemDB, from multiple chemical databases, comprising a total of 988 million molecules. In the original CReM framework, this fragment library consisted of over 40 billion fragments and exceeds 10 TB. In oCReM, the overall size is reduced by 85% to 1.5 TB.

This large reduction in fragment library size is crucial for molecular enumerations with such large fragment libraries, enabling CReM to explore ultralarge chemical spaces. We illustrate this point by (a) comparing the number of molecules enumerated for R-group and a core hop enumerations using ChEMBL22 and TandemDB, and (b) comparing the computational cost associated with such an R-group and core hop enumerations on a library as large as TandemDB using the original CReM framework and oCReM. The R-group enumeration is performed on a well-known Tyk2 inhibitor scaffold (Fig. 2C) [15], and the core hop enumeration is performed on the CDK2 inhibitor NU6094 (Fig. 2D) [13]. In the case of R-group enumeration, we observed approximately 50–100 times more molecules with varying R-groups and 20–80 times more molecules with different cores from using TandemDB compared to ChEMBL22, emphasizing the impact of large fragment libraries (Table 4).

A direct comparison of computational costs between CReM and oCReM reveals speed-ups of up to 85–95%, highlighting the impact of fragment library optimization on enumeration efficiency. We note that in this comparison, oCReM uses the Postgres format for the fragment libraries, whereas CReM uses SQLite. Given the comparable performance of CReM enumerations across both SQLite and Postgres formats for ChEMBL22, we anticipate similar behavior for the two formats of TandemDB as well.

The significance of the expanded, accessible chemical space afforded by TandemDB for the R-group enumeration on the TYK2 inhibitor scaffold is further illustrated in Fig. 3. For the enumeration with a context radius of 3, tSNE embeddings of compatible R-groups obtained from the ChEMBL22 and TandemDB libraries are shown in Fig. 3A and B, respectively. For ChEMBL22, all 38,788 molecules were included. For TandemDB, due to its large size, a structurally diverse subset of 30,000 R-groups, determined based on all-to-all Tanimoto similarity to the ChEMBL22 molecules, was selected from the 1.78M enumerated compounds (Table 4). As seen in the tSNE plots, TandemDB, which by design encompasses all R groups obtained from ChEMBL22, covers a significantly broader chemical space.

To evaluate the quality of the enumerated molecules, we went a step further and estimated their binding affinities using LigUnity, a foundation model for protein–ligand affinity prediction [16–18]. LigUnity is trained on a multi-task framework, leveraging both coarse virtual screening data and high-resolution binding affinity measurements, and can be fine-tuned with target-specific datasets. The predictive accuracy of LigUnity was demonstrated on a broad set of systems containing eight different benchmark datasets, where the model was shown to achieve state-of-the-art predictive performance across split-by-time, split-by-scaffold, and split-by-unit settings [18]. In this study, we employed the LigUnity model fine-tuned on internal binding affinity data derived from free energy perturbation (FEP) calculations of Tyk2 and CDK2 inhibitors [19].

The binding affinity predictions for TYK2 and CDK2 analogs generated from ChEMBL22 and TandemDB, as evaluated using the fine-tuned LigUnity model, are summarized in the “N mol (Good binders)” columns of Table 4, with the corresponding affinity distributions for TYK2 analogs shown in Fig. 3C and D. For the ChEMBL fragment libraries, when the number of generated molecules exceeded 50,000 (e.g., TYK2 radius 1 and 2 enumerations), a structurally diverse subset of 30,000 R-groups was selected based on all-to-all Tanimoto similarity. Predictions were performed using three independent random selections to ensure reproducibility. For TandemDB, in cases where more than 50,000 molecules were generated, a similarly diverse subset of 30,000 R-groups (for TYK2) or scaffolds(for CDK2) was selected based on all-to-all Tanimoto similarity relative to the ChEMBL molecules. Additionally, since FEP predictions, and consequently LigUnity’s performance, are most reliable for modeling smaller structural perturbations, affinity estimation was restricted to analogs differing by eight or fewer heavy atoms.

Given that the lead compounds from the inhibitor series based on the scaffolds shown in Fig. 2C and D, Compound 37 in Ref. [20] for TYK2 and Compound NU6102 in Ref. [21] for CDK2, are already highly potent, with single-digit nanomolar affinities ($\Delta G \sim -12$ kcal/mol), we defined “good binders” as analogs within a 10-fold range (1.4 kcal/mol) of the respective lead compound. Using this criterion, TandemDB libraries yield substantially more good binders than ChEMBL22. For example, in the TYK2 case with a context radius of 3, TandemDB produced 593 good binders compared to 76 from ChEMBL22, corresponding to nearly an eight-fold increase. The distribution of these compounds in t-SNE space further suggests that many of the good binders occupy regions of chemical space that are not accessible through ChEMBL22.

The docked 3D poses of the top five molecules with the highest predicted binding affinities are shown in Fig. 4 and summarized in Table S1. The docked poses of the enumerated molecules were generated using a constrained embedding approach, with the crystal structure of NU6094 serving as the reference. Notably, two of these molecules correspond to previously reported compounds from the same lead optimization series [20]. Specifically, Compound 1 and Compound 5 identified in our study are Compounds 30 and 39 in Ref. [20]. As shown in Fig. 4, these compounds establish novel interactions with the protein binding site residues that are likely the basis for their high predicted affinity.

**Table 4:** Enumeration statistics comparing ChEMBL22 and TandemDB fragment libraries

Mol	R	N mol		N mol (SA < 2.5)		N mol (Good binders)		Time (h)		Speed (%)
		ChEMBL	TandemDB	ChEMBL	TandemDB	ChEMBL	TandemDB	CReM	oCReM	
Tyk2	1	214,878	33,062,512	53,828	981,434	201	2142	109.2	11.3	89.6
	2	69,572	4,984,137	23,166	376,929	90	878	16.5	2.4	85.5
	3	38,788	1,786,673	15,049	210,325	76	593	5.9	0.8	86.4
CDK2	1	40,320	3,296,894	25,302	849,922	97	229	6.5	0.9	86.2
	2	3,365	61,091	2,478	32,871	2	41	0.2	0.03	85.0
	3	167	9,535	123	5,524	1	10	0.02	0.01	95.0

In both enumerations, fragment sizes were limited to 20 heavy atoms. All generated molecules were confirmed to be chemically valid using the *Chem.SanitizeMol()* function in RDKit. All enumerations were carried out on a single Intel(R) Xeon(R) CPU E5-2678 v3 @ 2.50GHz CPU

**Fig. 3:**
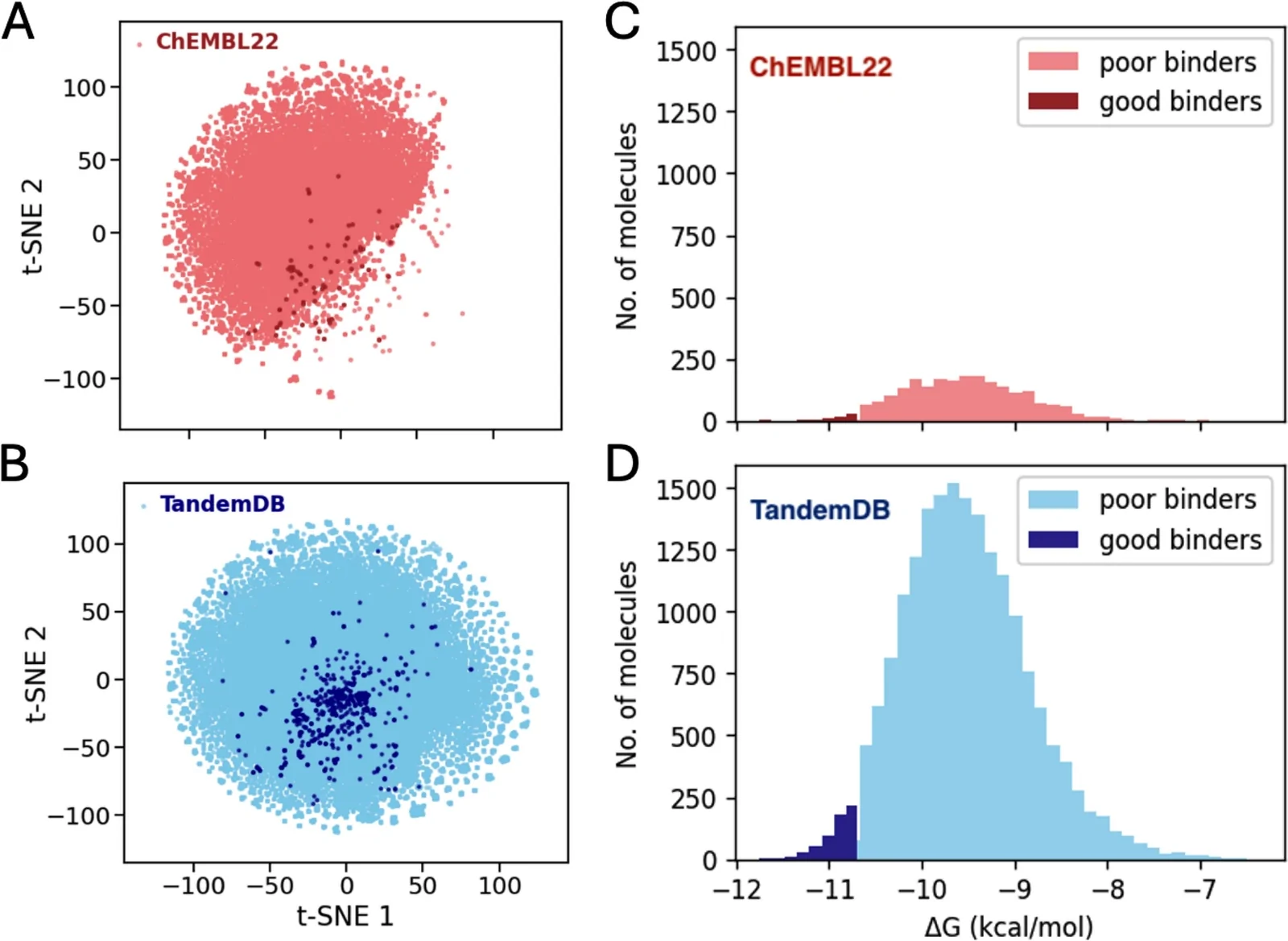
Expansion of the accessible chemical space through integration of TandemDB into CReM-based enumeration. Panels **A** and **B** are t-SNE embeddings of compatible R-groups derived from enumeration on a Tyk2 inhibitor scaffold shown in Fig. 2 (context radius = 3) using the ChEMBL22 and TandemDB fragment libraries, respectively. For ChEMBL22, all 38,788 molecules were included, whereas for TandemDB, a structurally diverse subset of 30,000 R-groups selected based on all-to-all Tanimoto similarity to the ChEMBL22 molecules (Table 4). The t-SNE embeddings for ChEMBL22 and TandemDB molecules were generated jointly and plotted separately, to ensure comparable axes across both panels. Panels **C** and **D** show the binding affinity (ΔG) distributions of the analogs enumerated with ChEMBL22 and TandemDB, respectively. In both cases, only analogs containing R-groups with eight or fewer heavy atoms were retained, resulting in filtered datasets comprising 2,301 molecules for ChEMBL22 and 18,789 molecules for TandemDB. Analogs that were predicted to have ΔGs less than −10.6 kcal/mol (within 10-fold of lead compound 36 in this series [20]) were classified as good binders and are shown in darker colors in both the distribution and t-SNE plots

**Fig. 4:**
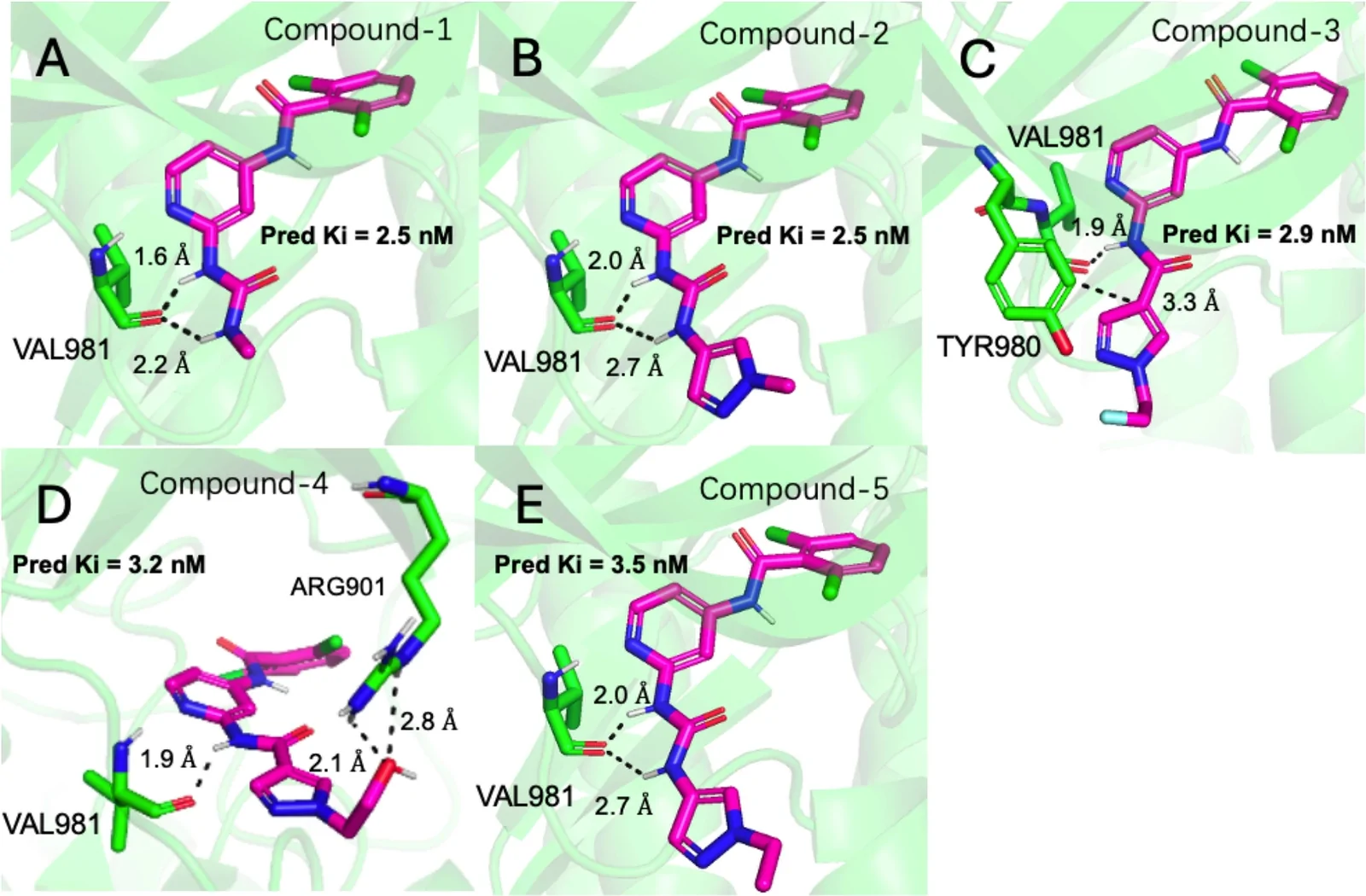
Panels A-E illustrate 3D poses of the top five CReM-enumerated molecules on a TYK2 inhibitor scaffold, with their predicted affinities and novel interactions from the added R-groups highlighted. All compounds form hydrogen bonds with the active-site valine (VAL981), while Compound 3 additionally exhibits a hydrophobic interaction with a nearby tyrosine (TYR980)

## Conclusion

We have introduced an optimized version of the CReM method, oCReM, that significantly reduces the size of the molecular fragment database and increases the speed of molecular generation, enabling the use of ultralarge fragment libraries on the scale of tens of billions of fragments. This optimization focuses on the architecture of the fragment database and the manner in which fragments are combined, while preserving all capabilities of the original CReM framework. We demonstrated how access to this expanded space afforded better small molecule compounds along multiple dimensions, especially that of protein-ligand potency as assessed by a machine learning model. oCReM extends the powerful CReM approach, allowing the incorporation of fragments from the rapidly growing chemical space of accessible molecules while retaining the power and flexibility of the original CReM approach to ideate novel molecules that are chemically valid and synthetically feasible.

## Supplementary Information

Supplementary material 1

## Data Availability

The code and data used in this manuscript are available at https://github.com/tandemai-inc/oCReM.
