# Supplementary material for: Expanding accessible chemical space for fragment-based enumeration by orders of magnitude through optimization of the Chemically Reasonable Mutations (CReM) framework
Source: J Cheminform. 2026 Aug 1;18:149. doi: 10.1186/s13321-026-01265-8 (PMC13721200; doi:10.1186/s13321-026-01265-8)
Supplement: Supplementary file 1 [file 13321_2026_1265_MOESM1_ESM.pdf]

Supporting Information

Compound	SMILES	Structure	Pred dG (kcal/mol)	Pred Ki (nM)	Expt Ki (µM)
Compound-1	CNC(=O)Nc1cc(NC(=O)c2c(Cl)cccc2Cl)ccn1	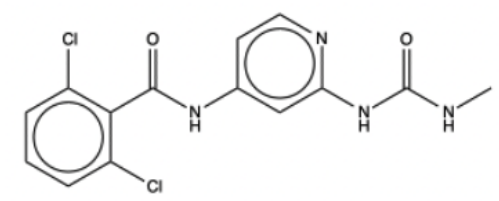	-11.75	2.5	1500
Compound-2	Cn1cc(NC(=O)Nc2cc(NC(=O)c3c(Cl)cccc3Cl)ccn2)cn1	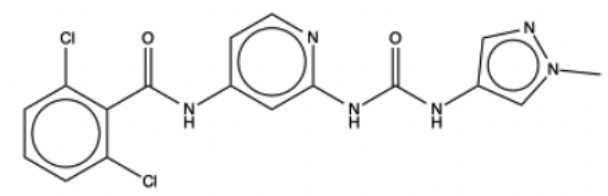	-11.73	2.5	-
Compound-3	O=C(Nc1cc(NC(=O)c2c(Cl)cccc2Cl)ccn1)c1cnn(CCF)c1	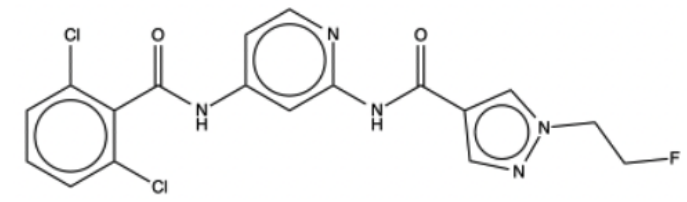	-11.68	2.9	-
Compound-4	O=C(Nc1cc(NC(=O)c2c(Cl)cccc2Cl)ccn1)c1cnn(CCO)c1	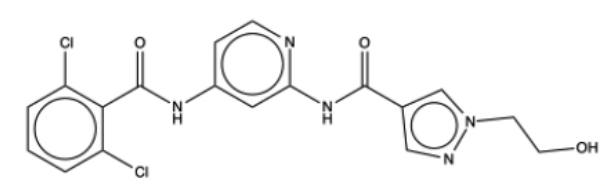	-11.60	3.2	-
Compound-5	CCn1cc(NC(=O)Nc2cc(NC(=O)c3c(Cl)cccc3Cl)ccn2)cn1	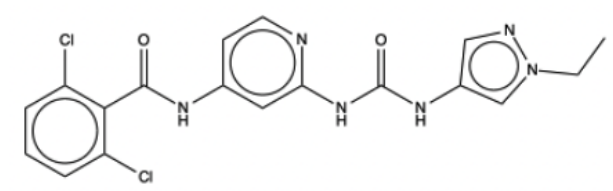	-11.55	3.5	19

Figure S1: Top 5 molecules with highest binding affinity scores obtained from an oCReM enumeration on a TYK2 inhibitor scaffold. Compounds 1 and 5 are known molecules previously reported in a TYK2 lead optimization study.[20]

Analysis of Minor Discrepancies Between CReM and oCReM Enumerations

While oCReM and CReM are expected to generate identical molecules when performing the same enumeration using the same fragment library, minor differences can occasionally arise in practice. To understand the origin of these discrepancies, we conducted a detailed analysis of all cases where the outputs of the two methods differed. This investigation revealed that the observed mismatches can be attributed primarily to two rare edge cases.

1. Loss of stereochemical information In certain cases, when the input SMILES contains double-bond stereochemistry and the modification site is adjacent to the stereogenic bond, CReM may lose stereochemical annotations during molecule generation.

Illustrative example

- Input SMILES:

  `C/N=C(\S)C1(C2CCNCC2)CCCS1`

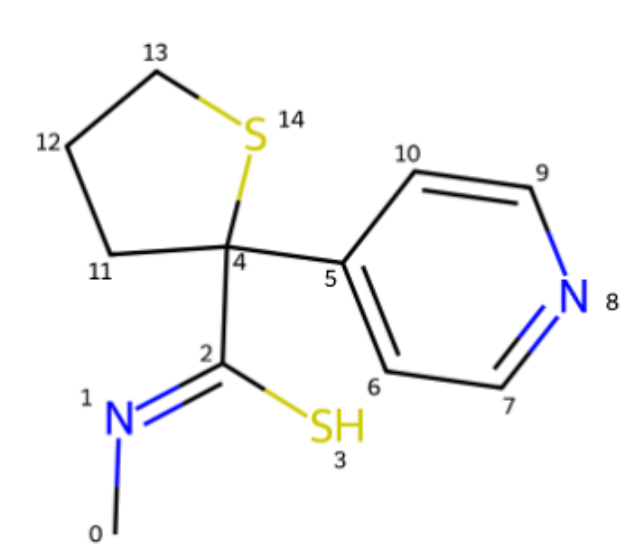

- GROW operation at carbon atom (ID: 0)
- CReM output:

  `CCCCCN=C(S)C1(C2CCNCC2)CCCS1`

  (double-bond stereochemistry lost)

- oCReM output:

  `CCCCC/N=C(\S)C1(C2CCNCC2)CCCS1`

  (double-bond stereochemistry preserved)

As a result, the same transformation may generate products that differ only in the retention of stereochemical annotations.

2. Ambiguity in reaction-site assignment In some GROW operations, the transformation rules used by CReM can match multiple chemically similar sites within a molecule. Consequently, products may occasionally be generated at positions other than the user-specified reaction site.

Illustrative example

- Input SMILES:

  `C1CCC2[NH]C(NN=C3CCCCCC3)NC2C1`

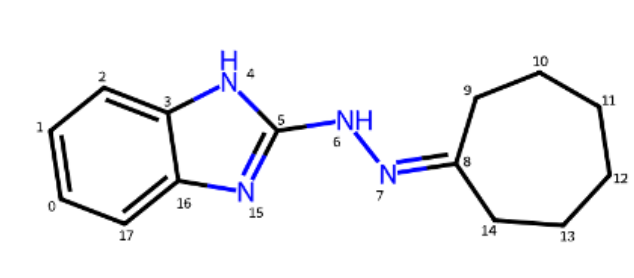

- GROW operation at carbon atom (ID: 17)
- Local chemical environment:

  `*:N:C1:C(-[*:1]):C:C:*:C:1:*`

- Retrieved fragment core:

  `C1=C([*:1])CCNC1`

For this example, oCReM generates a single product corresponding exactly to the requested growth site:

`C1=C(C2CCCC3[NH]C(NN=C4CCCCCC4)NC23)CCNC1`

In contrast, CReM generates two products:

1. The intended product corresponding to growth at atom C17.

2. An unintended product corresponding to growth at atom C2:

```
C1=C(C2CCCC3NC(NN=C4CCCCCC4)[NH]C23)CCNC1
```

Conversely, if growth is explicitly requested at atom C2, the local chemical environment becomes

```
*:[NH]:C1:C(-[*:1]):C:C:*:C:1:*
```

Under this environment definition, the fragment core

```
C1=C([*:1])CCNC1
```

is not retrieved from the fragment database, preventing generation of the corresponding product.

Taken together, these two edge cases: (i) occasional loss of stereochemical information and (ii) ambiguity in reaction-site assignment, can result in minor differences observed between CReM and oCReM enumerations. Importantly, these situations occur infrequently and have only a negligible impact on the overall agreement between the two methods. The consistency between CReM and oCReM remains exceptionally high across all benchmark datasets examined in this study.
